# The microbiota–gut–brain axis: pathways to better brain health. Perspectives on what we know, what we need to investigate and how to put knowledge into practice

**DOI:** 10.1007/s00018-021-04060-w

**Published:** 2022-01-19

**Authors:** Anirikh Chakrabarti, Lucie Geurts, Lesley Hoyles, Patricia Iozzo, Aletta D. Kraneveld, Giorgio La Fata, Michela Miani, Elaine Patterson, Bruno Pot, Colette Shortt, David Vauzour

**Affiliations:** 1 Cargill R&D Centre Europe, Vilvoorde, Belgium; 2 International Life Sciences Institute, European Branch, Brussels, Belgium; 3 Department of Biosciences, Nottingham Trent University, Nottingham, UK; 4 Institute of Clinical Physiology, National Research Council (CNR), Pisa, Italy; 5 Division of Pharmacology, Utrecht Institute for Pharmaceutical Sciences, Faculty of Science, Utrecht University, Utrecht, The Netherlands; 6 DSM Nutritional Products Ltd., Kaiseraugst, Switzerland; 7 IFF Health and Biosciences, Kantvik, Finland; 8 Yakult Europe BV, Almere, The Netherlands; 9 Ulster University, Coleraine, Co Londonderry, NI USA; 10 Norwich Medical School, Faculty of Medicine and Health Sciences, University of East Anglia, Norwich, UK

**Keywords:** Microbiome, Cognitive performance, Nutrition, Inflammation, Ageing, Mental health

## Abstract

The gut and brain link via various metabolic and signalling pathways, each with the potential to influence mental, brain and cognitive health. Over the past decade, the involvement of the gut microbiota in gut–brain communication has become the focus of increased scientific interest, establishing the microbiota–gut–brain axis as a field of research. There is a growing number of association studies exploring the gut microbiota’s possible role in memory, learning, anxiety, stress, neurodevelopmental and neurodegenerative disorders. Consequently, attention is now turning to how the microbiota can become the target of nutritional and therapeutic strategies for improved brain health and well-being. However, while such strategies that target the gut microbiota to influence brain health and function are currently under development with varying levels of success, still very little is yet known about the triggers and mechanisms underlying the gut microbiota’s apparent influence on cognitive or brain function and most evidence comes from pre-clinical studies rather than well controlled clinical trials/investigations. Filling the knowledge gaps requires establishing a standardised methodology for human studies, including strong guidance for specific focus areas of the microbiota–gut–brain axis, the need for more extensive biological sample analyses, and identification of relevant biomarkers. Other urgent requirements are new advanced models for in vitro and in vivo studies of relevant mechanisms, and a greater focus on omics technologies with supporting bioinformatics resources (training, tools) to efficiently translate study findings, as well as the identification of relevant targets in study populations. The key to building a validated evidence base rely on increasing knowledge sharing and multi-disciplinary collaborations, along with continued public–private funding support. This will allow microbiota–gut–brain axis research to move to its next phase so we can identify realistic opportunities to modulate the microbiota for better brain health.

## Introduction: a field of growing scientific interest

### The microbiota–gut–brain axis and the potential to support cognition and brain health

Does the gut hold the key to brain development and health? Through decades of research, scientists have established the strong connection between the gut and brain, modulated by neurons, neurotransmitters, hormones, and immune mediators (for details, we kindly direct readers towards extensive reviews [1–3]. More recently, focus has been extended to the role of the gut microbiota (referring to the trillions of microorganisms and viruses residing in the gut) [2, 4–6], creating considerable excitement with findings that suggest specific intestinal microorganisms (the greatest amount of information comes from studies of bacteria) may be associated with memory [7], learning [7], stress [8], and mood [6, 9, 10]—and even neurodevelopmental [11, 12] and neurodegenerative disorders [2].

Today, the so-called microbiota–gut–brain axis is an area of multi-disciplinary research that has captured international attention. Scientists specialised in neurology, endocrinology, immunology, microbiology, and bioinformatics have all found a niche worthy of exploration. Interest is such that international journals publish as many as 30 new studies a day related to this field.

While there is now considerable evidence that the microbiota–gut–brain axis plays an important role in mental and cognitive health, human clinical studies have as yet provided few clear answers to one burning question. How?

How does the gut microbiota influence brain development [13] and function [14]? Are brain disorders potentially shaped by the gut microbiota [15]? What role does diet play and what is its scope in influencing the microbiota–gut–brain axis [16, 17]? How do dietary supplements exert their apparent effect(s) on stress, mood, and cognition [18, 19]? What physiological mechanisms are at play [20]? And do alterations in microbiota–gut–brain interactions through life reflect the cause or symptom of an underlying brain condition [21]? Answering these questions is critical to harnessing the intestinal microbiota as a tool for ameliorating or preventing brain disorders, determining potential links with metabolic and cardiovascular diseases and for developing nutritional and therapeutic strategies that support and strengthen the brain health of the individual.

This perspective paper offers a short introduction to the microbiota–gut–brain axis, the knowledge and research so far and the considerable remaining gaps in the understanding of causes and mechanisms. Finally, the paper proposes how future meaningful progress can be made, which should benefit researchers active in fundamental and clinical gut–brain research from a multi or transdisciplinary perspective (including doctors and possibly patients/care takers), professionals in the mental health care, as well as research funders, food industry and investors. Once the mechanisms of gut microbiota modulation of brain health are unravelled, the potential for improving human quality of life and well-being is vast.

## The two-way street between gut and brain

### An introduction to microbiota–gut–brain communication, research, and potential therapeutic strategies

A ‘gut feeling’ or the sensation of ‘butterflies’ in the stomach are common illustrations of how a response in the brain is felt in the gut. Beyond that, microbiota–gut–brain interactions are much more complex to describe—as is abundantly clear from the intense research efforts to document them and propose links with brain development, physiology, function, and health.

As a highly complex community, the gut microbiota has a myriad of functions including education of the immune system, protection against pathogens, energy homeostasis and metabolite production. It is acknowledged that diet is a key determinant of composition of gut microbial populations and that it impacts on gut transit time and gut environmental conditions, and critically determines the supply of substrates for microbial growth [22, 23]. The gut microbiota has the potential to be both a mediator of the effect of diet and an effect modifier of the metabolic response to diet. In the case of the microbiota acting as a mediator, the dietary intervention acts directly on the microbiota, modifying the microbiota's composition and function. In contrast, as an effect modifier, the effect of diet on metabolism depends on the microbiota but the effect is not due to diet-induced changes in the microbiota. Thus, the gut microbiota is modifiable by diet and specific dietary components, and it plays a key role in shaping the composition and activity of the microbiota from birth, which impacts lifelong health [24–27].

In relation to brain development and brain health, up until now, many of the studies examining the microbiota–gut–brain axis have been performed in animal models; for example, germ-free, antibiotic-treated, genetically modified, or humanised mice, and behavioural models (for further details, we kindly direct readers towards extensive reviews [1]. Far fewer clinical studies have investigated whether the interactions observed in rodents are also observed in humans [6]. Due to a heavy reliance on association studies, there is still little evidence of the triggers and mechanisms linking the microbiota to gut–brain communication.

The extensive reviews by Cryan et al*.* [1] and Margolis et al*.* [6] are recommended reading for a detailed overview for the development of the microbiota–gut–brain axis, the pathways of communication involved, the modulating factors and the potential health implications [1, 6]. As the primary objective of this paper is to highlight the means for taking research to the next level of discovery, current microbiota–gut–brain axis knowledge is only briefly summarised here.

### Pathways for communication

At a fundamental level, the gut–brain axis is a bi-directional communication pathway composed of the central, enteric, and autonomic nervous systems and the hypothalamic–pituitary–adrenal (HPA) axis. The microbiota–gut–brain axis includes the gut microbes—comprising bacteria, viruses, fungi, and archaea—and their metabolites and by-products as factors in this bi-directional communication.

The vagus nerve, the immune and neuroendocrine systems, the neurotransmitters and metabolites along with the gut microbiota are currently the key pathways of interest in microbiota–gut–brain axis research [28].

#### The vagus nerve—the physical connection between brain and gut.

The tenth cranial nerve that extends from the brain to the abdomen is responsible for regulating internal organ functions such as digestion, heart rate and respiratory rate. Comprising efferent and afferent neurons, the vagus nerve carries motor signals between the brain and organs, including the intestinal cells, which are also subject to the influence of the gut microbiota. The brain is, in this way, able to ‘sense’ the environment in the gut [29, 30].

#### The immune system—firm roots in the gastrointestinal tract

Evidence of the immune system’s crucial role in gut–brain signalling is growing [31]. Today, it is widely recognised that most neurological conditions, including autism spectrum disorders (ASD), epilepsy, Alzheimer’s disease, Parkinson’s disease and cerebrovascular diseases, have low-grade systemic inflammatory components [32]. This low-grade inflammation is indicative of a malfunctioning immune response and dysbiotic microbiota.

Studies of germ-free mice and mice treated with broad-spectrum antibiotics have documented the gut microbiota’s involvement in intestinal immunity related to bacterial infections and inflammation [33]. Here, the microbiota was seen to regulate both innate and adaptive immunity—locally in the gastrointestinal (GI) tract and throughout the body. Scientists have similarly used such animal models to investigate the immunological effects of specific microbes in the gut microbiota.

From a brain health perspective, microbiota-immune interactions are of interest due to the systemic low-grade inflammation often seen in neurodegenerative, neuropsychiatric, and metabolic disorders. For example, there have been extensive studies of the causal role of the microbiota in inflammatory bowel disease (IBD), which is associated with an increased susceptibility to Parkinson’s disease [33, 34].

#### The neuroendocrine system—gut hormones and the regulation of well-being

Recent studies suggest that gut hormones are involved in the physiological processes that lead to disorders such as anxiety and depression—with indications that mood disorders and obesity often co-exist [35]. Scientists focus increasingly on the ability of the microbiota to modulate gut hormones and, through that, their potential to regulate mood.

Increasing evidence supports the concept of bi-directional communication between the neuroendocrine system and gut microbiota. Disturbances in both systems have been associated with disorders such as depression and irritable bowel syndrome [36]. Findings further indicate that the gut microbiota can activate the HPA axis [36]—one of the body’s major neuroendocrine systems that controls responses to stress and is involved in regulating, for example, mood and emotions [37] and the immune system [38].

A growing body of research suggests that a number of neurotransmitters function as hormones and vice versa. Dopamine and serotonin, for example, are known to have hormonal properties [39]. Although these hormone-like neurotransmitters are not solely produced in the gut, the gut microbiota is thought to play a role in their modulation.

#### Neurotransmitters and metabolites

Evidence from animal studies suggests the host’s physiology is affected in various ways by the ability of gut microorganisms to produce and metabolise a range of neurotransmitters, although this remains to be documented in human subjects [13]. In the context of the microbiota–gut–brain axis, noteworthy neurotransmitters include dopamine, serotonin, noradrenaline, and gamma-aminobutyric acid (GABA). The neuroactive amino acids tyramine and tryptophan, short-chain fatty acids (SCFA), and bile acids are other molecules of interest.

##### GABA

GABA is believed to have a role in behaviour, cognition and the body’s response to stress, anxiety and fear [40], while low GABA levels are associated with psychiatric illnesses, including schizophrenia, autism and depression [41]. Although the regulatory importance of the microbiota is not yet fully mapped, studies of germ-free animals suggest that the microbiota influences circulating GABA levels [42]. GABA is also produced by some *Lactobacilli* [43] and specific strains of *Bifidobacterium* [13, 44].

##### Serotonin and tryptophan

Much research has linked the microbiota with serotonin regulation in the gut [45, 46]. Serotonin is involved in mood, cognition, sleep, and appetite control [46]. Today, selective serotonin reuptake inhibitors (SSRI) are commonly prescribed treatments for depression as they increase the level of available serotonin in the brain [47]. Studies also focus on the amino acid tryptophan as the sole precursor of serotonin. It has been proposed that gut microbiota may influence tryptophan uptake and, in that way, serotonin synthesis [47].

In addition, 90% of tryptophan in the intestinal tract is metabolised along the kynurenine pathway. Of particular interest are the neuroactive metabolites quinolinic and kynurenic acids that affect the enteric nervous system (ENS) and central nervous system (CNS) (for review see [48, 49]).

##### Dopamine

Dopamine is a major neurotransmitter associated with the brain’s reward system and is a precursor for epinephrine, also known as adrenaline, and norepinephrine, which contributes to arousal and alertness as well as behaviour and cognition [13]. Disorders associated with dopamine deficiency include addiction, schizophrenia, and Parkinson’s disease. Research suggests that certain bacteria produce [13] or metabolise [50] dopamine.

##### SCFAs

The SCFAs propionate, butyrate and acetate are metabolites mainly produced and regulated by the bacterial fermentation of complex plant-based polysaccharides in the gut [51]. In recent years, research has explored the potential role of SCFAs in gut–brain communication with and across the blood–brain barrier (BBB) [52] and in supporting BBB integrity—a progressively leaky BBB being seen in Alzheimer’s disease [15].

Studies have led to a wide range of findings that connect butyrate, for example, with memory, cognition, mood, and metabolism [53]. Acetate has been associated with appetite regulation [54], and propionate may be involved in protecting against type 2 diabetes and obesity and reducing stress behaviours [55].

#### Gut microbiota—the omnipresent factor, modulated by diet

Research has repeatedly revealed new aspects of the microbiota’s contribution to gut–brain crosstalk, beginning with maternal nutrition [56] and the colonisation of the infant gut at birth [15]. It is also known that age, gender, genetics, environmental factors, geography, disease, exercise, fasting [57] and diet influence the microbiota’s composition—diet and nutritional status being among the most influential factors [28, 58]. Recent reviews give a comprehensive overview of the role of diet in shaping the gut microbiota [59–61]. The gut microbiota itself can influence dietary preferences via the mesocorticolimbic system, responsible for the hedonic response to food intake [62].

Greater knowledge of the gut microbiota represents exciting possibilities to track changes in microbiota composition, activity, and behaviour in relation to the development and progression of brain disorders. Another promising avenue of exploration is the modulation of the gut microbiota by specific dietary components such as probiotics, prebiotics, postbiotics, synbiotics, and parabiotics. Such work could lead to novel therapeutic strategies, fuelled by so-called microbiotic medicinal products (MMPs) [63].

### The potential for nutritional and therapeutic strategies

Research has established many links, associations, and hypotheses about the lifelong influence of the gut microbiota on brain health. Underlining this critical role, one review ranks the gut microbiota as the fourth key factor in early-life programming of brain health and disease, alongside prenatal and postnatal environment, and host genetics [64]. The scientific challenge is to identify opportunities to alter and fine-tune the microbiota and, through that, enhance human health and well-being.

To this end, animal and human clinical trials have explored dietary supplementation with pro-, pre-, syn- and postbiotics, omega-3 polyunsaturated fatty acids [64] and phytochemicals, such as polyphenols, which may act as prebiotics [65]. High-fibre diets—promoting SCFA production by the gut microbiota—are a promising intervention to overcome maternal-obesity-induced impairment of cognitive and social functions [66]. Faecal microbiota transplants are another potential therapeutic opportunity, having already been shown to influence hedonic food intake in mice [62]. Here, important regulatory differences apply whether developing strategies for clinical therapies or foods.

#### Regulation of stress, mood, and anxiety

Research has associated the gut microbiota with a range of stress- and mood-related conditions [8]. In relation to stress, several clinical studies have linked probiotic and prebiotic supplementation with a positive outcome [67–69]. The majority of mood and anxiety studies, on the other hand, have relied on pre-clinical animal models [8]. Healthy mice that received a probiotic formulation with *Lactobacillus rhamnosus,* for example, were seen to perform best in tests designed to provoke anxiety, depression, and stress [70].

Clinical trials have often produced conflicting results. While some have observed a significant reduction in stress and anxiety following probiotic intervention with *Lactobacillus* (sensu lato) and *Bifidobacterium* strains [58], others have not [70]. Reviews of clinical trials found probiotics had a limited effect on psychological outcomes—although this could be partly explained by an incomplete evidence base along with a large heterogeneity in the population, cognitive tests, and interventions [70]. Another study reported a positive probiotic effect on mood and anxiety in patients with IBD [71].

#### Implications for autism spectrum disorder

The microbiota has been demonstrated to have a clear role in autism spectrum disorder (ASD). One study has observed how the transplantation of microbes from a human diagnosed with ASD induced-like behaviour in mice [72]. Conversely, several clinical studies of ASD have found that microbiota modulation through antibiotic, prebiotic and probiotic and faecal transplantation treatments can improve social behaviour [73–76]. Researchers have further reported a reduction in anxiety behaviour, hyperactivity and defiance behaviours [73].

Other findings show that children diagnosed with ASD are four times more likely to have GI symptoms, including inflammation and abdominal pain [73] and that faecal transplantation may have long-term beneficial effects on intestinal and behavioural symptoms [76].

#### Learning and memory

A number of studies have explored the relationship between the gut microbiota and the development of learning and memory systems in childhood [77]. This has led to a growing appreciation that sensitive periods of development occur across the microbiota–gut–brain axis.

From animal studies, there is increasing evidence that changes in the gut microbiota alter performance in relation to visual-spatial learning and memory tasks [78]. Although there are still few human data, one study has associated microbial diversity with cognitive functioning in infancy [77].

A new approach to cognitive development research is required, including the microbiota–gut–brain axis as a peripheral force among the complex biological systems that act on behaviour. By improving understanding, this may lay the foundation for innovative therapies for learning and memory disorders [77].

#### Cognitive performance and age-related disorders

Many scientists now believe in the close relationship between microbial diversity and healthy ageing. Studies in mice have shown that faecal microbiota transplantation can correct age-related defects in immune function [33]—and that a similar transplant from aged to young mice has a detrimental impact on key functions of the CNS [79, 80]. These and other findings highlight the importance of the microbiota–gut–brain axis during ageing and raise the possibility that a ‘young’ microbiota may maintain or improve cognitive functions in life’s later years [81, 82].

Neurological research suggests the microbiota also play a role in neurodegenerative diseases [83]. This supports the idea that an ageing gut microbiota could be linked to immune and neuronal dysfunction in Parkinson’s and Alzheimer’s disease. Indeed, studies of faecal microbiota transplants in transgenic mouse models point to a causal relationship between intestinal microbiota, protein aggregation and cognitive problems [84–86]. More studies are necessary to confirm this.

### Knowledge with potential

Whether changes in the microbiota are key to detecting and understanding the physiological processes that lead to brain disorders is still unknown. But the possibilities are undeniable. Research has uncovered positive indications that therapeutic interventions may have a beneficial impact, for example in neurodevelopmental disorders, such as ASD, and age-related neurodegenerative disorders [15]. And there is every reason to be optimistic about the potential to reduce stress and anxiety. The task now is to overcome the barriers to further discovery.

## Shortfalls and challenges—the bottlenecks to progress

### The need for more knowledge and comprehensive study designs

Research in the microbiota–gut–brain axis has reached a crossroad. The gut microbiota’s omnipresence and overlapping influence on physiological systems has made it progressively challenging to discuss individual aspects of the microbiota–gut–brain axis in isolation—underlining the need for a multi-disciplinary, multi-system research approach to uncover the mechanisms and opportunities for improving human quality of life and well-being, as is being done for metabolic diseases [87, 88]. Multidomain interventions combining diet, with other health-promoting lifestyle approaches, have been demonstrated to be effective strategies as they target endogenous and environmental factors (such as genetics, age, diet, and lifestyle) that modulate the gut microbiota activity and composition, underlining enormous variability between individuals [89, 90].

Consequently, while many of the tools and methodologies in use until now have significantly advanced our knowledge and understanding of the role of the microbiota–gut–brain axis in brain health and disease, the large majority of studies to date have been limited to animal models and have mostly been observational in a clinical setting. There are still many unanswered questions within the field which require more clarity in order to drive further meaningful progress towards microbiota-targeted strategies for improving brain health. Some of the gaps in current knowledge are fundamental and must be bridged by skilful scientific investigation.

### Understanding changes and mechanisms

The characteristics and function of a ‘healthy’ gut microbiota are still unknown. Although studies have frequently documented a reduction in functional diversity and compositional alterations in relation to a variety of disorders [61], there is as yet little understanding of how the microbiota changes over time and may reflect the impending onset of disease. Recent data from more than 9000 adults of different ages show that, as individuals age, the gut microbiome becomes increasingly unique, increasingly different from others, starting in mid-to-late adulthood. A better understanding of this phenomenon may open the way to an improved understanding of what is a ‘healthy ageing microbiota pattern’ [91]. Similarly, there is lack of knowledge about disease biomarkers and whether they may be reversed through treatment or dietary interventions. Several systematic reviews and meta-analyses, albeit with different search criteria, have investigated the effects of probiotics, prebiotics, and even fermented foods on symptoms of depression, anxiety, and mood, as well as on cognition. Interestingly, while the majority of studies did conclude there were some positive effects of dietary interventions or supplements on depression and anxiety symptoms [18, 19, 70, 92], others concluded that the data to support the role of dietary interventions on mood and cognitive function were insignificant [93, 94]. In addition, some studies reported that targeting the gut microbiome to alleviate symptoms of anxiety and depression were more pronounced in clinical patient populations compared with healthy adults [95]. Finally, most studies did suggest that additional double-blind, randomised, placebo controlled clinical trials in clinical populations are warranted to further assess efficacy.

Numerous association and correlation studies have identified links between the gut microbiota and the CNS [96–99]. Further targeted studies are required to identify and confirm the mechanisms of action in humans. Complex gaps in existing knowledge include:

- The immunological effects of specific microbes in the human gut microbiota and their role in neurodevelopmental, neurodegenerative, and neuropsychiatric disorders.
- A precise mapping of microbiota-regulated neurotransmitters in human subjects, the hormonal properties of these neurotransmitters and the mechanisms by which they activate the HPA axis.
- How microbial by-products, such as SCFAs, branched-chain fatty acids, methylamines, and peptides, influence brain function in tandem with immunological and neurological signalling molecules.
- The contribution of specific microbes to brain development during early life.

### Few and varied clinical studies

Intervention studies in humans and pre-clinical studies in humanised mice and rats are a fundamental requirement. In the early days of this research field, most research was limited to in vitro or pre-clinical studies, and there was a high prevalence of review articles and meta-analyses of the microbiota compositions [96, 100–103]. Since, clinical intervention studies have been performed more frequently although often characterised by a low number of human subjects and short timeframes [94, 104].

As typical in nutritional intervention studies, the non-standardised approaches often used means that the authors of review articles frequently struggle to find suitable clinical studies for meaningful comparisons. Wide variations in test subjects, cognitive and mental test designs, intervention formulations and the filtering of data stand in the way of general conclusions—with many studies being low on statistical power [94].

Overall, clinical studies are held back by a lack of disease- and microbiota-specific biomarkers, absence of clinically relevant behavioural phenotypes and poor tools for cohort stratification. Still, over the last year a number of meta-analyses have appeared which show a moderately positive evaluation on the use of psychobiotic [104] interventions for anxiety [105], schizophrenia [106] or cognitive functions [107, 108], pointing to the diversity and complexity of—and the numerous confounding factors that may affect—the gut microbiota [21, 109].

Furthermore, when trying to establish cause and consequence relation, it might also be important to better understand the effects of traditional drugs, including psychotropics, on the microbiota and the potential health consequences [110].

A general tendency to conduct pre-clinical and clinical studies within the silos of individual disciplines also compounds these limitations and, at the same time, rules out the opportunities created by multi-disciplinary collaboration. The time has clearly come for a new approach.

## Beyond hypotheses to validated nutritional and therapeutic strategies

### Practical proposals for moving microbiota–gut–brain axis research forward

**Fig. 1:**
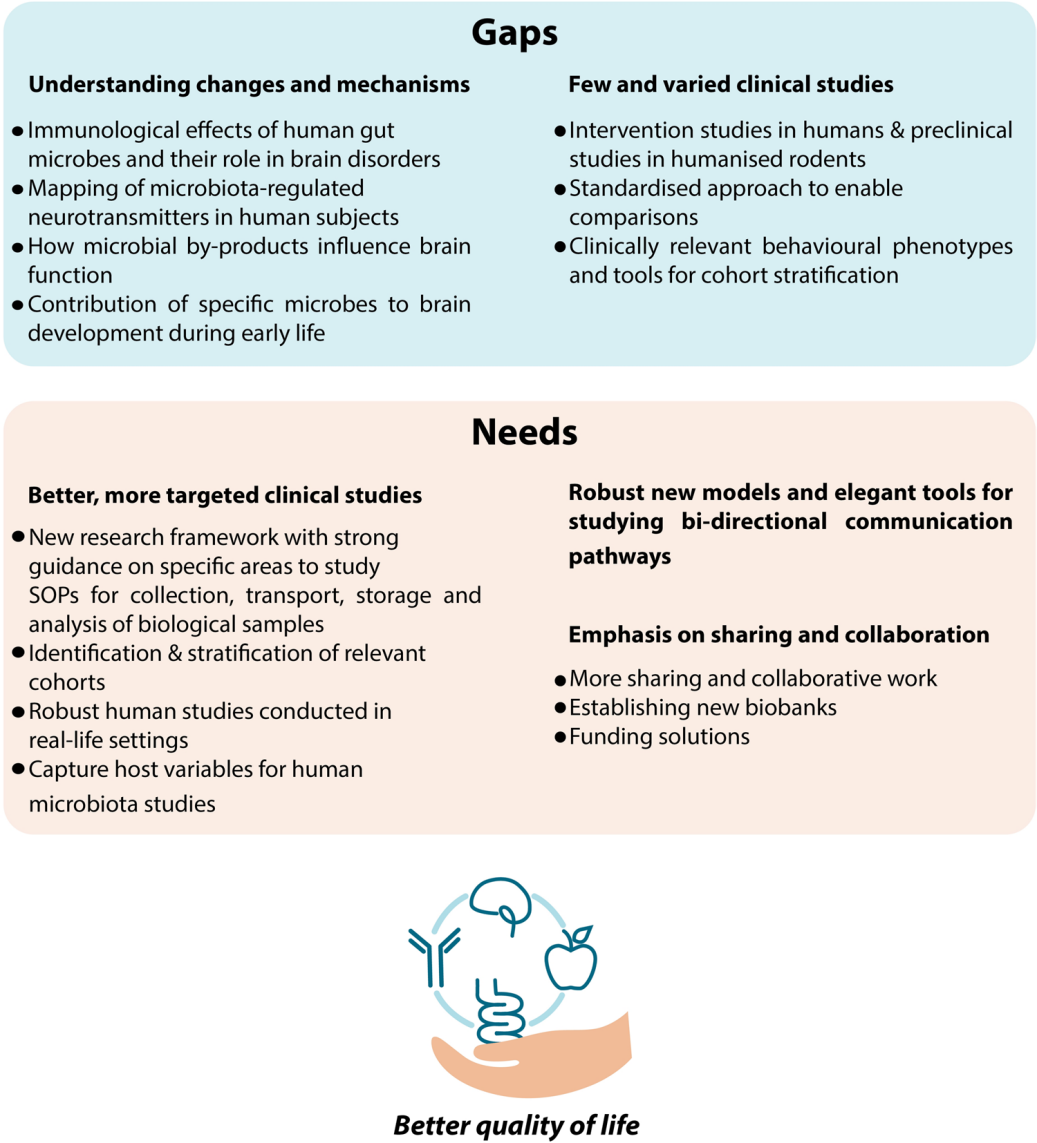
Key gaps and needs in microbiota–gut–brain axis research on the journey towards nutritional and therapeutic strategies for improved quality of life

As the microbiota–gut–brain axis continues to attract scientific attention, a whole-system, multi-disciplinary approach is necessary to progress from hypotheses to validated therapeutic strategies of benefit to brain health. Scientists have successfully documented countless associations between the gut microbiota and brain disorders. However, correlation does not equal causation. The next step is to understand the mechanisms behind those associations and how they are influenced by dietary habits, lifestyle, and genetic risk factors. This will require new methods, skills, and collaborations. An overview of the gaps and needs is represented in Fig. 1.

### More targeted, gold standard clinical studies with reproducible results

Experiences so far highlight the need to rethink and redesign the approach to clinical studies in a way that facilitates the integration of standardised methods and models from all fields of study related to the microbiota–gut–brain axis. The emphasis on ‘standardised’ is important. In this context, human clinical studies should be robust, employing a design that includes randomisation, controlled with a suitable placebo, and conducted at least double-blind. Clinical trials should always be conducted in accordance with the Declaration of Helsinki [111] and the guidelines for Good Clinical Practice (GCP) [112] to ensure ethical and scientific quality requirements are followed throughout the study design, conduct, recording of information, and reporting of data. Compliance with this standard ensures not only that the rights, safety, and well-being of trial subjects are protected, but also that the data is credible. Finally, only by conducting repeated studies that provide comparable and reproducible results will it be possible to build a critical mass of scientific evidence to drive real progress.

A new research framework should include strong guidance on specific areas of the microbiota–gut–brain axis to investigate, which biological samples to collect and the biomarkers or surrogate biomarkers to measure—with regard to sampling and analysis, the NIH Human Microbiome Project website already provides some guidance [113]. Standard operating procedures should also be established for the collection, transport, storage, and analysis of biological samples and for the sequencing and filtering of data, reducing the variables that can influence study outcomes. Equally important are the identification and stratification of relevant cohorts to support cross-study comparisons and consolidate research findings (e.g., The Quadram Institute website released for best practice in microbiome research [114]).

Robust human studies must be conducted in real-life settings using calibrated dietary habit assessments and validated test methods to investigate potential windows for nutritional strategies [115]. At present, studies of dietary habits rely on subjects to provide data by filling out food frequency questionnaires, 24-h recalls, food checklists, diet histories, and food diaries which require large and complicated data analyses and experienced dieticians or nutritionists to accurately extrapolate the data [116]. To improve the quality of these data, there is a need to replace self-reporting with new and emerging objective tools. The emergence of food intake biomarkers holds great promise for nutrition research in this regard [117, 118]. Another possibility is to recruit subjects who share the same household or live in a care home, for example, where they tend to eat the same foods. One recent study by Valles-Colomer et al*.* assessed gut microbiota compositional covariation with quality-of-life indicators and depression in the Belgian Flemish Gut Flora Project population cohort [119]. While *Faecalibacterium* and *Coprococcus* were consistently associated with higher quality of life indicators, both of these genera were depleted in depression and inflammatory bowel disease. Interestingly, *Coprococcus* and *Dialister* decreased with depression. These results were validated in other large microbiome cohorts. To investigate the link between microbial neuroactive capacity with quality of life and depression, the authors constructed the first catalogue of gut microbiota neuroactive potential using a module-based analytical framework. Specific covariations were discovered between pathways of neurotransmission, mental quality of life and specific genera such as *Coprococcus* [119]. New investigative tools such as the gut–brain module analysis of faecal metagenomes described by Valles-Colomer et al*.* could provide greater insight into the associations between pathways regulating brain health and function, the gut microbiota, and symptoms of mood disorders commonly found across different population cohorts. Clinical studies of the role of microbiota in disease must account for the natural variations in microbiota composition from one individual to the next. Age, sex, body mass index, medications, and lifestyle are among the host variables that confound microbiota analyses and limit the capacity to draw valid conclusions. For example, research has shown that patients with depression have an altered gut microbial profile compared with healthy adults [119–122]. However, each study describes unique microbial changes in these patients due to huge inter-individual microbial differences in the general human population. This variability between studies makes it extremely difficult to interpret whether the microbial changes described are a hallmark of depression or whether they are unique to one individual study. Indeed, this is an important limitation to consider before drawing conclusions on the role of the gut microbiome in mental disorders such as depression. Furthermore, investigations into the gut microbial profile of patients with depression do not indicate whether these changes are causal to disease state or consequential of disease. In studies of personalised interventions based on intestinal microbiota composition and activity, an unhealthy diet, for example, may negate the potential beneficial effects of a dietary supplement. Nutrition, physical activity, psychological and physical stress, sleep restrictions, socioeconomic status, antibiotics use, exposure to pets, noise, and temperature have been all reported to associate with changes in human microbiota [123–125]. It is, therefore, essential that human microbiota studies capture such host variables to secure reproducible evidence about the relationship between specific gut microorganisms and biomarkers of disease [126]. The appropriate timing of an intervention is an additional factor to account for, considering that the impact of lifestyle and environments may vary along the lifespan. Intervening during sensitive time-windows, e.g., when microbiota and brain are still developing and their plasticity is high, may increase the likelihood of a persistent effect. Studies in the first 1000 days of life indicate that exposure to antibiotics [127, 128], pets, siblings [129], specific maternal intakes (sweeteners [130]) and environmental toxicants [131] affecting the infant’s microbiota are likely targets. On the other hand, since diet and lifestyle are such strong drivers of microbiota composition and activity [132], this opens the possibility to help patients to take their own responsibility to improve their brain health. Indeed, there is accumulating evidence in nutritional psychiatry regarding the importance of diet for realising mental health [133]; however, the causational role of the gut microbiome needs to be established. This challenge cannot be tackled by observational studies and interventional studies examining the effects of dietary and/or lifestyle changes as well as interventions with nutraceuticals. It needs to be designed in a different way, because the classical double-blinded approach does not work. A combination of alternative interventional study approaches, such as cross-over studies (for example [134], or citizen science (for example [135]) combined with mechanistic studies using new models and tools might be the way forward.

### Robust new models and elegant tools

Future progress further relies on the development of new models and elegant tools for studying bi-directional communication pathways. While animal models have proven invaluable in establishing the current knowledge base, it is inescapable that the gut microbiota of rodents is substantially different from that of humans. To overcome this limitation, there is a need for robust and reliable humanised rodent models [136].

From the perspective of in vitro models, three-dimensional brain and gut organoids and advanced co-culture systems including the ENS, vagus nerve and the BBB provide alternative methods for investigating realistic conditions for unravelling the mysteries of microbiota–gut–brain mechanisms [137, 138]. Used in combination with models for digestion, such organoids and co-cultures could form in vitro workflow models for studying the gut–brain axis in context. A number of so-called organ-on-a-chip in vitro models have already been developed for this purpose, though they still have limitations [139].

Great opportunities also lie in the development of methods that track, for example, how neurotransmitters travel from the gut through the BBB in response to neuroinflammatory processes. Some of this methodology is becoming available, with human brain imaging representing a possibility to track the influence of microbiota on neurotransmission [13]. Metabolomic, metaproteomic and metagenomic analyses and gut biopsies are other possible methodologies.

Many research studies today involve statisticians from their inception to assure the quality of the study’s design. Computational and data scientists are similarly vital to maximise the value of research through comprehensive data analysis. Specialised computer programmes are already able to provide next-level precision when generalising and stratifying results in relation to specific population groups, such as those at risk of brain disorders [140].

Machine learning technology will become increasingly essential to improving the efficiency and accuracy of study findings. Indeed, bioinformatics holds the key to integrating large, multi-dimensional datasets and, from that, gaining a better understanding of their clinical significance. At the current pace of technological development, it is now possible to imagine the potential of such tools to identify high-risk patients at an early stage, determine which microbial/immunological imbalances may cause such risks and suggest possible interventions to mitigate them [141].

### An emphasis on collaboration

More sharing and collaborative work is essential to extract maximum knowledge from available data and build a truly validated evidence base. This requires the establishment of new biobanks to facilitate the sharing of material from human and animal studies. Deep phenotyping databases, standardised data formats [142] and new methodologies for preserving microbiome samples [143] are essential for such biobanks to play a meaningful role. By the same token, in vitro models must become more easily available for use across labs.

The competition for funding is one explanation for the low level of scientific collaboration to date. However, a number of programmes and initiatives are, today, moving research in this direction. Within Europe, they include the Community Research and Development Information Service (CORDIS) [144], which gathers and disseminates results from projects funded by the EU’s framework programmes for research and innovation.

One such project is the five-year multi-centre GEMMA project funded by the EU’s Horizon 2020 programme [145]. Launched in January 2019, GEMMA explores interactions between the gut microbiome, metabolome, epigenome, and immune function to discover useful biomarkers for early diagnosis of autism, along with potential targets for preventive therapies [146]. Other examples are the ONCOBIOME [147] and MICROB-PREDICT [148] projects, funded by Horizon 2020 to investigate the microbiome’s role in cancer development and chronic liver disease, respectively.

Organisations such as the International Life Science Institute Europe (ILSI Europe) [149] and the International Scientific Association for Probiotics and Prebiotics (ISAPP) [150] bring together academic and industrial scientists involved in basic and applied research across multiple disciplines. Their purpose is to promote progress in the field by supporting scientific integrity and transparency, harmonising scientific efforts, and providing guidance for collaborative and multi-disciplinary research.

ISAPP is setting an excellent example. Each one of its objectives is relevant to the progress of microbiota–gut–brain axis research at large and the ultimate development of dietary strategies where the gut microbiota is the primary target.

## The dream destination—improved quality of life

### The potential of the microbiota–gut–brain axis through future nutritional and therapeutic interventions

The microbiota–gut–brain axis represents an intricate network of systems which scientists are only beginning to understand. Given this complexity, the nutritional and therapeutic strategies with the best chances of success are likely to be those aimed at improving human quality of life. Some are even already on the market, including foods and supplements that promise to improve mood, sleep, or cognitive performance. The evidence behind some of these claims is, however, still in question. Very recently though, the European Food Safety Agency (EFSA) approved *Akkermansia muciniphila* as a novel food [151].

By expanding knowledge, scientists have recognised the potential to achieve much more. Although the prevention of brain disorders may remain out of reach for the foreseeable future, the mapping of healthy microbiota and communication pathways could enable their early prediction. The first signs of neurodegenerative conditions such as Alzheimer’s and Parkinson’s disease, for example, are known to develop many years before diagnosis. Imagine if it were possible to slow neurodegenerative processes by altering the microbiome.

A similar scenario is imaginable for children with ASD. What if dietary influences on the gut microbiota could both relieve GI irritation and calm anxiety and hyperactivity? And what if it were possible to complement drug and psychiatric therapy for schizophrenia with targeted foods such as probiotics?

These are, perhaps, realisable dreams. Over the past few years, they have inspired a growing number of scientists to found start-up companies that are now investigating small molecule therapeutics for treating neurological and other disorders through microbiome modulation. Private investors often support their clinical research.

Scientists have documented many links between the microbiota, gut, and brain. The time has come to dig even deeper through integrated, multi-disciplinary research—aimed at understanding microbiota–gut–brain mechanisms and identifying true opportunities to adapt and adjust the microbiota for better brain health through life. Continuous investment from the public and private sector is vital to keep up the momentum.

## Data Availability

Not applicable.
